# Life table study of sublethal concentrations of emamectin benzoate against *Spodoptera frugiperda* (Lepidoptera, Noctuidae)

**DOI:** 10.1093/jisesa/ieaf014

**Published:** 2025-02-17

**Authors:** Chengyu Chen, Yiting Tang, Yunxia Zhao, Xuefeng Zhang, Kai Zhang

**Affiliations:** Plant Protection center, Huaiyin Institute of Agricultural Sciences of Xuhuai Region in Jiangsu, Jiangsu Academy of Agricultural Sciences, Jiangsu Province 223001, China; China National Agricultural Science Observation and Experiment Station of Huai’an, Jiangsu Province 223001, China; Plant Protection center, Huaiyin Institute of Agricultural Sciences of Xuhuai Region in Jiangsu, Jiangsu Academy of Agricultural Sciences, Jiangsu Province 223001, China; China National Agricultural Science Observation and Experiment Station of Huai’an, Jiangsu Province 223001, China; Plant Protection center, Huaiyin Institute of Agricultural Sciences of Xuhuai Region in Jiangsu, Jiangsu Academy of Agricultural Sciences, Jiangsu Province 223001, China; China National Agricultural Science Observation and Experiment Station of Huai’an, Jiangsu Province 223001, China; Plant Protection center, Huaiyin Institute of Agricultural Sciences of Xuhuai Region in Jiangsu, Jiangsu Academy of Agricultural Sciences, Jiangsu Province 223001, China; China National Agricultural Science Observation and Experiment Station of Huai’an, Jiangsu Province 223001, China; Plant Protection center, Huaiyin Institute of Agricultural Sciences of Xuhuai Region in Jiangsu, Jiangsu Academy of Agricultural Sciences, Jiangsu Province 223001, China; China National Agricultural Science Observation and Experiment Station of Huai’an, Jiangsu Province 223001, China

**Keywords:** emamectin benzoate, *Spodoptera frugiperda*, sublethal effects, population growth parameters, two-sex life table

## Abstract

The fall armyworm, *Spodoptera frugiperda* J.E. Smith (Lepidoptera: Noctuidae), is a well-known agricultural pest in North and South America and has invaded Africa, the Far East, and Australia in the past decade. To investigate the integrated management of *S. frugiperda*, the sublethal impacts of emamectin benzoate were studied. Leaf-dipping bioassays were used to investigate the effects of sublethal (LC_10_ and LC_30_) concentrations of emamectin benzoate on *S. frugiperda* demographic parameters, and data were interpreted based on the age-stage, two-sex life table theory. The results showed that the developmental time of larvae was prolonged while the fecundity decreased after sublethal concentration treatment. Furthermore, the intrinsic and finite rates of increase, as well as the net reproductive rate, significantly declined following LC_30_ concentration exposure, whereas the average generation time was extended in comparison to the control group. The intrinsic rate of increase (*r*_m_) dropped to 0.14 (LC_10_) and 0.13 (LC_30_)/day, compared to the control group (0.18/day). The net reproductive rate (*R*_0_) dropped from 775.40 to 303.10 and 193.30 after the LC_10_ and LC_30_ concentration treatment, respectively. In this study, sublethal concentrations of emamectin benzoate adversely affected the developmental time, fecundity, and life table parameters of *S. frugiperda*.

## Graphical Abstract

**Graphical Abstract:**
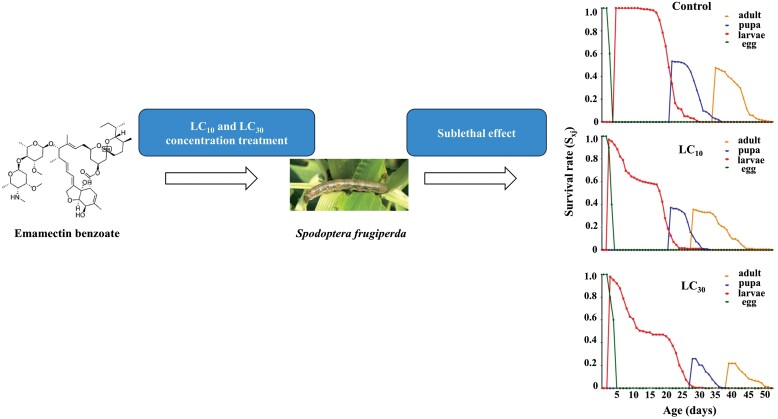

## Introduction

The fall armyworm, known as *Spodoptera frugiperda* (J. E. Smith) (Lepidoptera: Noctuidae) is indigenous to tropical and subtropical regions of America. It is recognized as a significant agricultural pest by the Food and Agriculture Organization (FAO) due to its detrimental impact on various crops, including corn, wheat, sorghum, and sugarcane, resulting in substantial economic losses (Malo et al. 2013, Tay et al. 2023, Wang et al. 2023, 2024). Its destructive potential extends to 353 plant species from 76 families (Sisay 2019). The fall armyworm has rapidly spread across Africa and Asia in recent years. China experienced severe losses in corn production following the invasion of the fall armyworm in December 2018. (Wu et al. 2019, Jing et al. 2020). The larvae exhibit a preference for tender leaves of the plant and demonstrate a robust reproductive capacity, with an average fecundity of approximately 1500 eggs and a maximum of 2000 eggs (Yan et al. 2022).

Up to now, insecticides are still the main way to control *S. frugiperda* (Guo et al. 2022, Chang et al. 2023). Emamectin benzoate is a macrolide disaccharide derived from abamectin B1, a potent semisynthetic antibiotic insecticide, and its mode of action involves causing a continuous flow of chlorine ions in the GABA and H-Glutamate receptor sites (Hüter et al. 2011). It was reported emamectin benzoate had good toxic effect against many kinds of lepidopteran pests, such as *Helicoverpa armigera* (Hübner) (Lepidoptera:Noctuidae), *Spodoptera littoralis* (Boisd) (Lepidoptera:Noctuidae), *Spodoptera exigua* (Hübner) (Lepidoptera:Noctuidae), *Plutella xylostella* (Linnaeus) (Lepidoptera: Plutellidae) (Tang et al. 2021, Ye et al. 2022, Khalifa et al. 2023). With no exception, studies reported that the emamectin benzoate had a good control effect on *S. frugiperda* in the corn field (Babendreier et al. 2020, Chen et al. 2022, Patil et al. 2022). The application of insecticides results in pests experiencing varying concentrations of these chemicals over time, which subsequently induces a series of biological and ecological effects. Consequently, both the acute lethal effects and the sublethal effects of pesticides should be evaluated (Řezáč et al. 2021, Lu et al. 2023a). For instance, the pupation rate of *S. frugiperda* was significantly reduced following exposure to the LC_30_ concentration of chlorantraniliprole, dinotefuran, and beta-cypermethrin, respectively (Wu et al. 2022). Therefore, emamectin benzoate might have a sublethal impact on *S. frugiperda* when used in field condition.

Life table is an effective method to evaluate population dynamics and to forecast pest outbreaks (Zhang et al. 2014). Numerous studies have utilized the age-stage, two-sex life table theory to assess how insecticides impact insect survival and reproduction (Huang et al. 2012, Iqbal et al. 2022). This kind of research can be extended to populations with both sexes and age-stage structures, incorporating variations in preadult development, which will enhance the precision of survival and reproductive rate curves. Different sublethal concentrations may cause different sublethal effects on pests, this research employed life table analysis to examine how sublethal doses of emamectin benzoate (LC_10_ and LC_30_) impact the population parameters of newly emerged *S. frugiperda* larvae. The effect of sublethal of emamectin benzoate on *S. frugiperda* was studied, which could provide theoretical basis for sustainable management and scientific application of emamectin benzoate.

## Material and methods

### Insect cultures

A laboratory strain of *S. frugiperda* was originally obtained from Huaian, Jiangsu Province, China in 2020, and was reared on the artificial diet (mainly contain soybean flour, wheat germ meal, yeast powder) at 25 ± 1 °C with 60 ± 5% relative humidity, and a 14-h light/10-h dark cycle without pesticide exposure (Chen et al. 2022).

### Chemical reagent

Emamectin benzoate with 83.5% purity was purchased from Qilu Synva Pharmaceutical co., LTD., Jinan, Shandong Province. Triton X-100 and acetone were obtained from Sinopharm Chemical reagent limited company.

### Bioassays

The leaf-dipping bioassay method was used to test the effects of *S. frugiperda* on newly hatched larvae (Chen et al. 2022). The stock solution was prepared by dissolving emamectin benzoate in acetone. The stock solution was serially diluted with distilled water containing 0.1% (v/v) Triton X-100 (0.001, 0.002, 0.004, 0.008, 0.016 mg/l, respectively). Corn leaves (2–3 cm) were sliced and immersed in the test solution for 30 s with light stirring, air-dried at room temperature, and then placed in 12-well tissue-culture plates covered with 2% (w/v) agar and filter paper. Four replicates (approximately 30 larvae per replicate) of 5 concentrations were used. The blank control group was treated with leaves dipped in distilled water containing 0.1% (v/v) Triton X-100 and 1% (v/v) acetone. This experiment repeated for 3 times, and mortality was assessed 24 h after insecticide application. Larvae were considered dead if they showed no movement when touched with a damp brush.

### Sublethal effects of emamectin benzoate on biological parameters and life cycle

The newly hatched larvae were collected and treated with emamectin benzoate at 2 sublethal (LC_10_ and LC_30_) concentrations with the bioassay method described above. A life table study was conducted with 200 larvae per treatment placed on fresh corn leaves in individual glass tubes (height: 9 cm, diameter: 1.5 cm), and kept in the growth chamber at 25 ± 1 °C with 60 ± 5% relative humidity, and a 14-h light/10-hour dark cycle. The study included 3 treatments (LC_10_, LC_30_, and control), with each larva treated as an individual replicate (Chi and Yang 2003, Schneider et al. 2009). Daily records were kept on the survival and development of the larvae. Fresh corn leaves were provided to the larvae. When the larvae turned into pupae, transferred to fresh glass tubes. One male and one female adult were paired after emergence and subsequently placed into separate plastic containers for egg-laying (a plastic cup 5 cm wide and 8 cm tall, with a cotton ball soaked in 10% honey water). The adults were checked daily for oviposition, then the newly laid eggs were transferred to new glass tubes. The survival rate and fecundity of each individual were calculated until they died.

### Data analysis

GraphPad 7 was utilized to examine the data. The data obtained from the bioassay experiments were subjected to probit analysis using the SPSS program (version 19). The unprocessed data for each individual was examined using the age-stage two-sex life table theory (Chi 1988, Chi et al. 2020). Excel 2019 was utilized to record the developmental time, longevity, and fecundity. Statistically significant differences were calculated by one way ANOVA and Tukey’s test with GraphPad 7 (*α* = 0.05).

From the raw data, the age-stage specific survival rate (*s*_*xj*_, represents the probability that a newborn egg will survive to age × and stage j), age-stage specific fecundity (*f*_*xj*_), age-specific survival rate (*l*_x_= $\overset{k}{\underset{j=1}{\sum }}s_{xj}$), and age-specific fecundity (*m*_x_= $\frac{\overset{i}{\underset{j=1}{\sum }}s_{xj}{f_{x}}_{j}}{\overset{i}{\underset{j=1}{\sum }}{s_{x}}_{j}}$) were calculated (Zhao et al. 2016, Wu et al. 2022).

The net reproductive rate (*R*_0_) was calculated as follows: *R*_0_ = $\overset{\infty }{\underset{x=0}{\sum }}l_{x}m_{x}$

The mean generation time (*T*) was calculated as follows:0 *T* = $\frac{\overset{\infty }{\underset{x=0}{\sum }}xl_{x}f_{xj}}{R_{0}}$

The intrinsic rate of increase (*r*_m_) was calculated using the following equation: *r*_m_ = $\frac{\ln R_{0}}{T}$

The population double time (*t*) was calculated using the following equation: *t* = $\frac{ln2}{r_{m}}$

The finite rate of increase (*λ*) was calculated as $\lambda =e^{rm}$

GraphPad 7 was used to construct survival rate, fecundity, and life expectancy curves.

## Results

### Toxicity of emamectin benzoate on the larvae first instar of *S. frugiperda*

The toxicity of emamectin benzoate against the 1st instar larvae of *S. frugiperda* was given in Table 1 (*χ*^2^=3.94, df = 3, *P* = 0.27). The LC_10_ and LC_30_ values were 0.0024 (0.0018-0.0030) and 0.0044 (0.0036-0.0052) mg/L, respectively.

**Table 1.** Toxicity of emamectin benzoate on the larvae first instar of *Spodoptera frugiperda*

Concentration mg/L		Slope ± SE	*χ* ^2^	df	*p*
LC_10_ (95% confidence limit)	LC_30_ (mg/L) (95% confidence limit)				
0.0024(0.0018–0.0030)	0.0044(0.0036–0.0052)	2.88 ± 0.32	3.94	3	0.27

### Life table of *S. frugiperda* exposed to sublethal concentrations of emamectin benzoate

The development time, longevity, and fecundity of *S. frugiperda* in the presence of sublethal concentrations of emamectin benzoate were studied (Table 2). The larval stage of *S. frugiperda* after the LC_10_ and LC_30_ concentration treatment (23.3 days and 24.0 days, respectively) was significantly longer than that of the control (21.8 days). However, the pupal stage of *S. frugiperda* in the control group was longer than that of the LC_30_ treatment group.

**Table 2.** Life tables of *Spodoptera frugiperda* exposed to sublethal concentrations of emamectin benzoate

Stages	Control	LC_10_	LC_30_	*P*	*F*	*df*
Larval (days)	21.8 ± 0.2b(*n* = 95)	23.3 ± 0.3a(*n* = 72)	24.0 ± 0.3a(*n* = 54)	<0.01	24.04	220
Pupal (days)	9.7 ± 0.1a(*n* = 75)	9.4 ± 0.1ab(*n* = 68)	9.2 ± 0.1b(*n* = 47)	<0.01	5.04	189
Mean longevity of adult (days)	13.5 ± 0.3a(*n* = 70)	12.9 ± 0.4a(*n* = 65)	13.0 ± 0.5a(*n* = 45)	0.43	0.84	179
Fecundity (eggs/♀)	577 ± 73.9a(*n* = 36)	249 ± 28.6b(*n* = 25)	328 ± 41.2b(*n* = 20)	<0.01	8.68	80

The different lowercase letters within each row indicate significant differences by Tukey’s test (ANOVA, *P* < 0.05).

Emamectin benzoate’s sublethal effects on population parameters were summarized in Table 3. Following the application of emamectin benzoate, the intrinsic and finite rates of increase, net reproductive rate, and mean generation time were notably impacted at the LC_30_ concentration level (Table 3). The intrinsic growth rate decreased to 0.13, compared with the control group (0.18 day, *P* = 0.02). The finite rate of increase (*λ*) exhibited a pattern comparable to the intrinsic rate of increase. The net reproductive rates (*R*_0_) for control, LC_10_, and LC_30_ treated larvae were 775.40, 303.10, and 193.30 offspring per individual, respectively, showing a significant reduction following emamectin benzoate exposure (*P* = 0.02). The mean generation time (*T*) for the treated group exceeded that of the control group.

**Table 3.** The sublethal effects of emamectin benzoate on *Spodoptera frugiperda* population parameters

Population parameters	Control	LC_10_	LC_30_	*P*	*F*	*df*
Intrinsic rate of increase (*r*_m_)	0.18 ± 0.01a	0.14 ± 0.01ab	0.13 ± 0.01b	0.02	6.61	10
Finite rate of increase (*λ*)	1.19 ± 0.01a	1.15 ± 0.01ab	1.14 ± 0.01b	0.02	6.55	10
Net reproductive rate (*R*_*0*_)	775.40 ± 178.1a	303.10 ± 122.5ab	193.30 ± 53.5b	0.03	5.94	10
Mean generation time (*T*)	37.29 ± 0.37b	40.15 ± 0.23a	39.40 ± 0.41a	<0.01	16.07	10
Population double time (*t*)	3.99 ± 0.23b	5.05 ± 0.34ab	5.42 ± 0.43a	0.04	4.86	10

The different lowercase letters within each row indicate significant differences by Tukey’s test (ANOVA, *P* < 0.05).

**Fig. 1:**
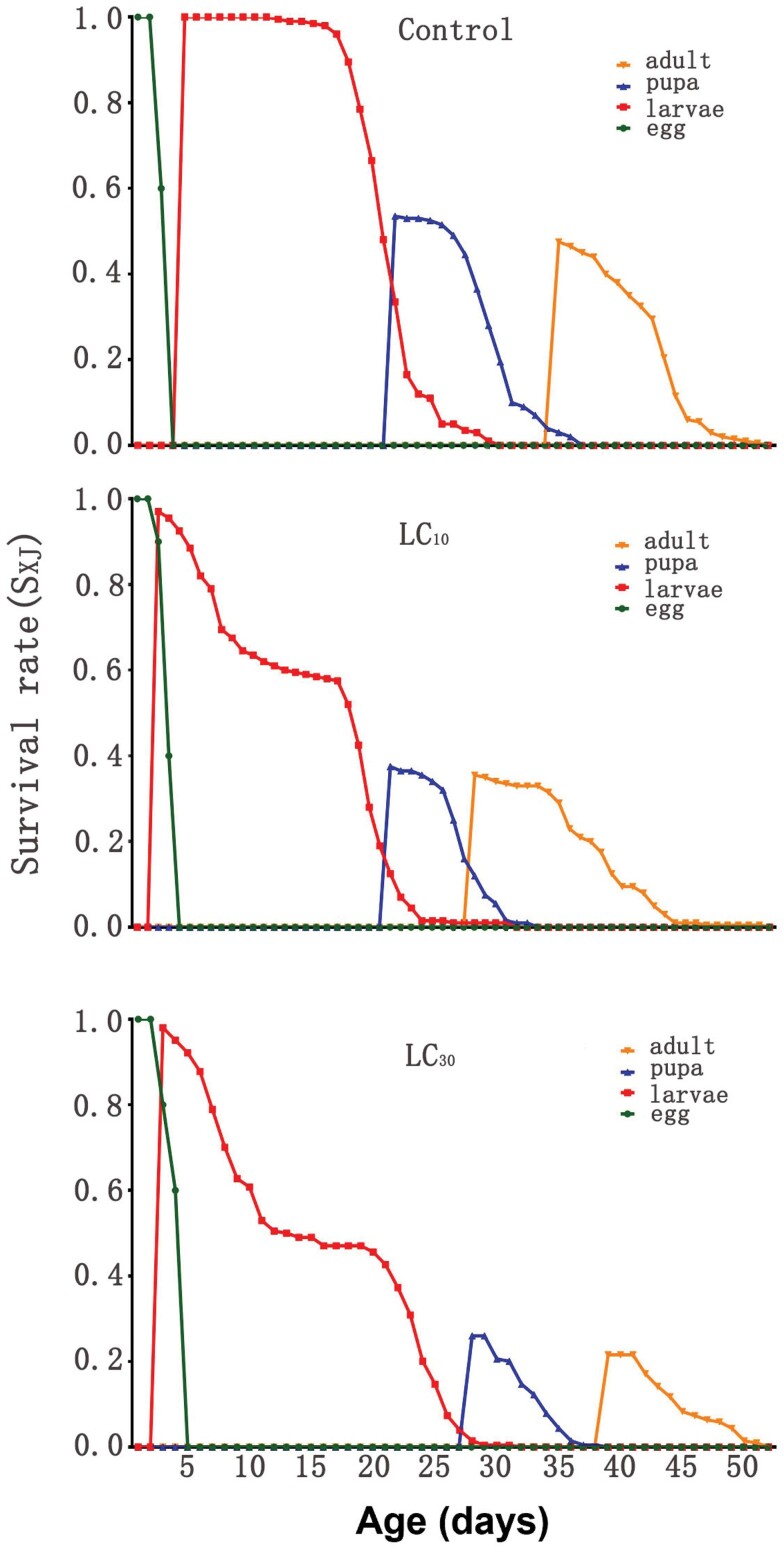
Age-stage specific survival rate (*s*_*xj*_) of *Spodoptera frugiperda* treated with sublethal concentrations of emamectin benzoate

As a result of the differences in individual developmental rates, curves with obvious overlaps were observed (Fig. 1). In comparison to the control, larvae numbers declined after LC_10_ and LC_30_ treatments. The overall development duration for larvae treated with LC_10_ concentration was extended compared to the control group.

**Fig. 2:**
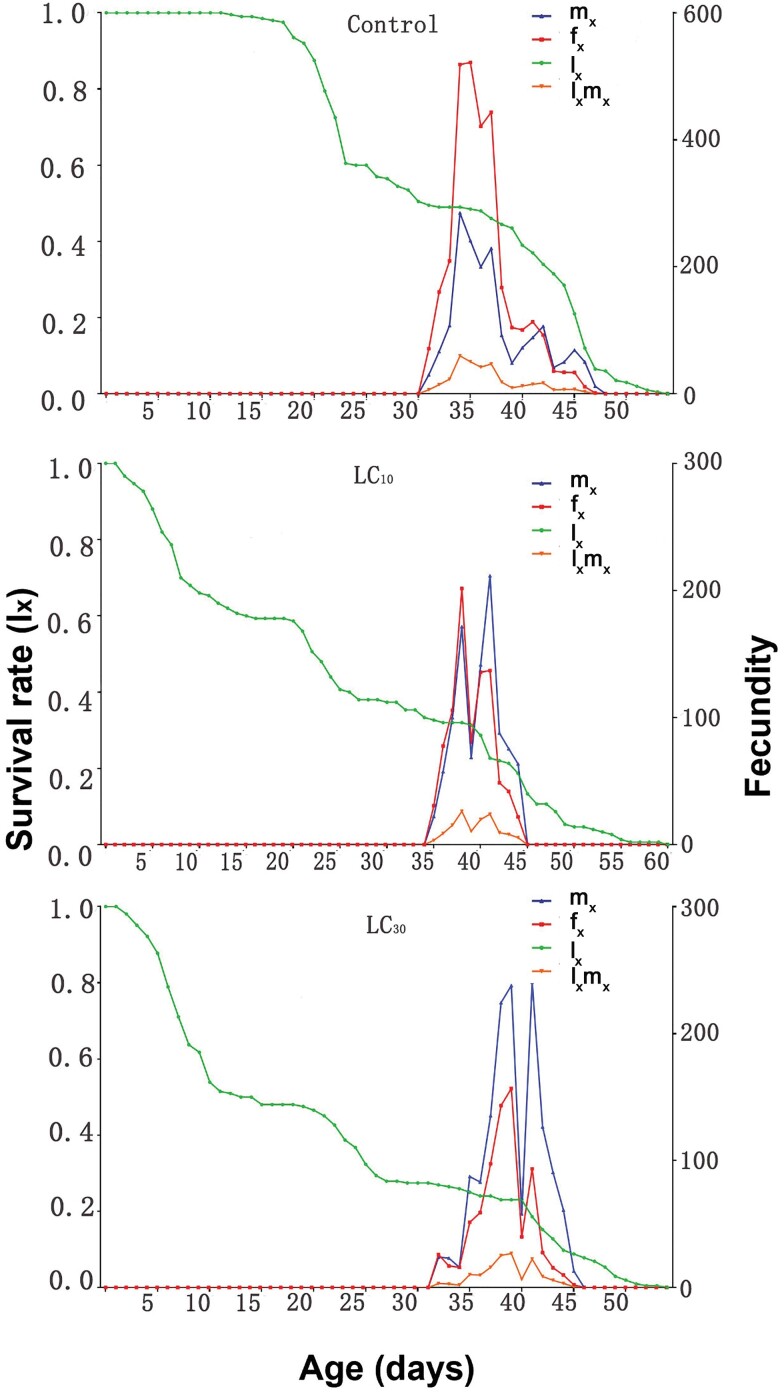
Age-specific survival rate (*l*_*x*_), female age-specific fecundity (*f*_*x*_), age-specific fecundity (*m*_*x*_), and age-specific maternity (*l*_*x*_*m*_*x*_) of *Spodoptera frugiperda* treated with sublethal concentrations of emamectin benzoate

In addition to the measurement of survival rates (*l*_*x*_), fertility rate (*f*_*x*_) among females, population fertility rate (*m*_*x*_), and maternity rate (*l*_*x*_*m*_*x*_), we also assessed the age-specific birth rate (*l*_*x*_) as depicted in Fig. 2. The control group exhibited higher peaks in *f*_*x*_ compared to the LC_10_ and LC_30_ treatment groups. The value of *l*_*x*_*m*_*x*_ is influenced by *l*_*x*_ and *m*_*x*_, with the maximum *l*_*x*_*m*_*x*_ values observed at 37, 39, and 40 days for control, LC_10,_ and LC_30_, respectively.

**Fig. 3:**
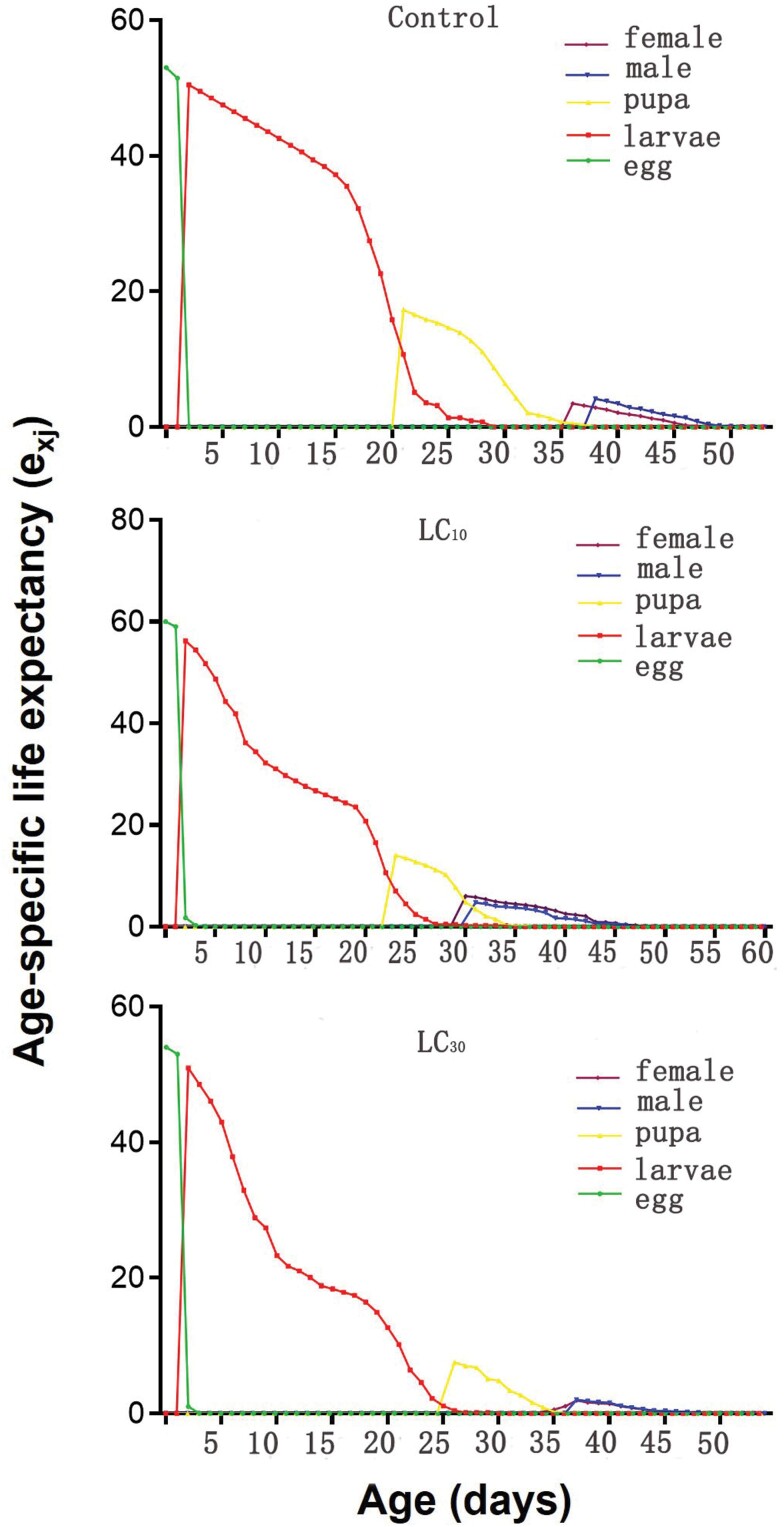
Age-stage-specific life expectancy (*e*_*xj*_) of *Spodoptera frugiperda* treated with sublethal concentrations of emamectin benzoate

**Fig. 4:**
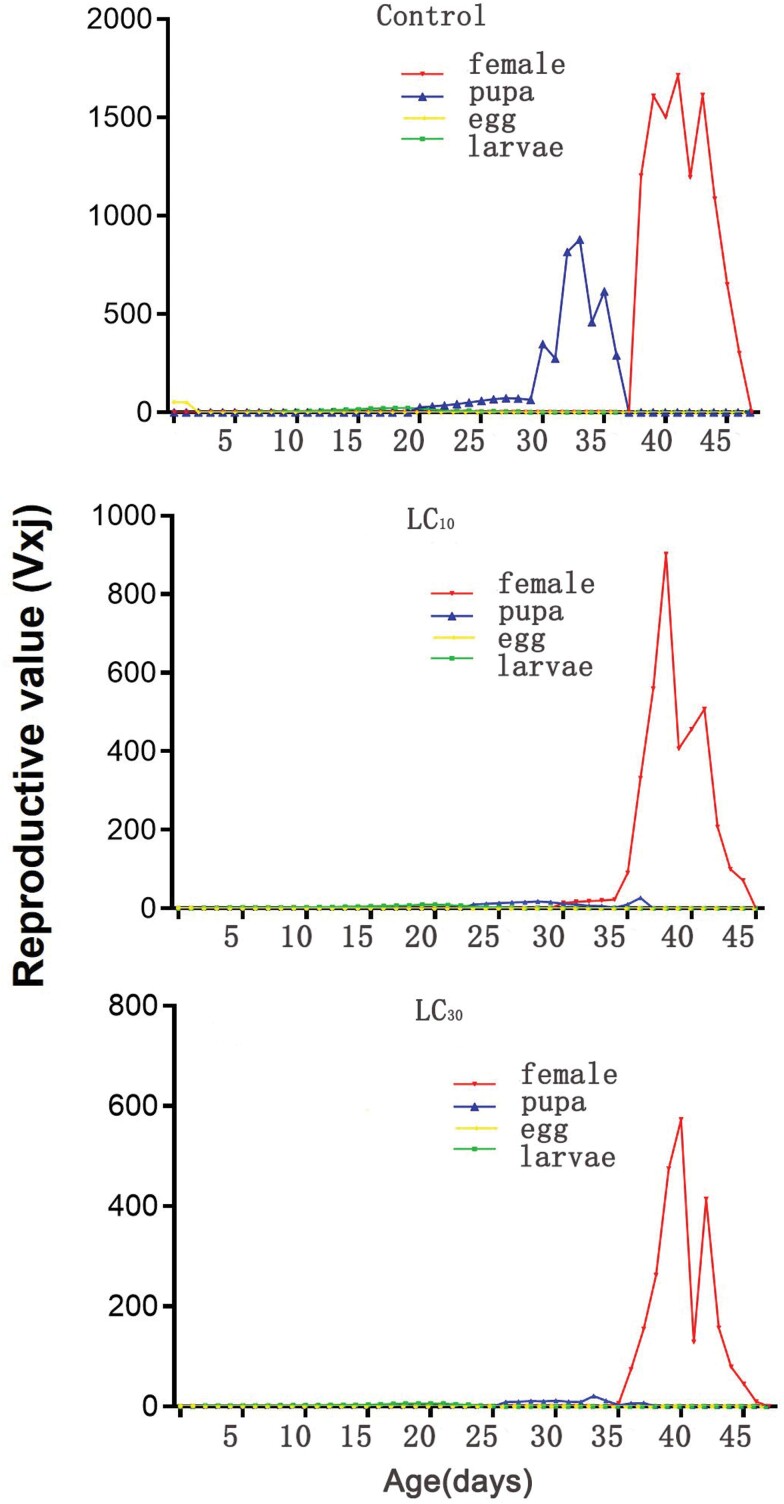
Age-stage reproductive value (*v*_*xj*_) of *Spodoptera frugiperda* treated with sublethal concentrations of emamectin benzoate

The age-stage specific life expectancies (*e*_*xj*_) represent the anticipated duration of an individual’s life at a given age × and stage *j*. In comparison to the control group (53 days), the sublethal concentrations (LC_10_ and LC_30_) emamectin benzoate treatments exhibited longer expected lifespans for newly emerged eggs, specifically 60 days and 54 days, respectively (Fig.3). Figure 4 shows the curves (*v*_*xj*_) representing the assessment of the contribution to future offspring from age ×  to stage *j*. The female reproductive value peak exhibited no significant change. Nevertheless, the reproductive peak of the *S. frugiperda* offspring in the LC_10_ and LC_30_ treatment groups reached 902 eggs at 39 days and 573 eggs at 41 days after treatment, respectively, both of which were lower than the reproductive peak observed in the control group. The control group reached a reproductive peak of 1714 eggs at 42 days after treatment.

## Discussion

It has been reported that embamectin benzoate sublethal doses significantly affected insect development, growth, and reproduction, such as *H. armigera* (Hübner), *P. xylostella* (Dong et al. 2011, Liu et al. 2022). In this study, age-stage 2-sex life tables were employed to examine the impact of sublethal concentrations (LC_10_ and LC_30_) of emamectin benzoate on various population parameters of *S. frugiperda*. The results showed that the pupal development time was significantly shorter after LC_30_ concentration treatment, this was same with that the pupal development time was shorter in *Thrips hawaiiensis* (Thysanoptera: Thripidae) after treated with sublethal concentrations (LC_10_ and LC_20_) of emamectin benzoate (Chen et al. 2023). In this study, the fecundity of *S. frugiperda* was significantly decreased by emamectin benzoate LC_10_ and LC_30_ concentration treatment. This is similar to what was reported earlier by Lu et al. (2023b). These outcomes provide evidence that sublethal doses of emamectin benzoate effectively inhibit the growth of *S. frugiperda* populations.

The evaluation of the total impact of insecticides on insect populations has been recommended by Stark and Banks (2003) through the utilization of population parameters. In this study, the intrinsic rate of increase (*r*_m_), finite rates of increase (*λ*) and net reproduction rate (*R*_0_) were significantly decrease with LC_30_ concentration, this is somewhat consistent with the previous report by Abbas (2023). These decreases were attributed to the decline in survival rate (*l*_*x*_) and fecundity observed in *S. frugiperda* upon treatment with sublethal concentrations of emamectin benzoate, as depicted in Figs. 1 and 2. The inclusion of survival, development, and reproduction in the calculation of the intrinsic rate of increase (*r*_m_) makes it a valuable parameter for studying populations (Maia et al. 2000). A decrease in the survival rate (*l*_*x*_) can result in a corresponding decrease in the intrinsic rate of increase (*r*_m_), which can have detrimental effects on the population. This phenomenon has also been observed in *S. littoralis* when exposed to sublethal concentrations of emamectin benzoate (Mokbel and Huesien 2020).

According to previous discussions, species-specific factors and environmental influences can contribute to the variance in survival rates among insect species or within the same species under different conditions. The *s*_*xj*_ value, a metric for measuring survival rate, was found to decrease as the exposure to emamectin benzoate increased (Fig. 1). This observation aligns with previous findings on the sublethal effects of emamectin benzoate on *Panonychus citri* (Khan et al. 2021).

A controlled laboratory study can provide valuable insight into the dynamics of a species’ population even though insects rarely undergo constant conditions in the natural world. In this study, sublethal concentrations of emamectin benzoate adversely affected the population parameters of *S. frugiperda*. There would be a reduction in pesticide use, control costs, and environmental pollution as a result. In integrated management of *S. frugiperda*, sublethal doses of emamectin benzoate showed distinct advantages over more acute insecticide treatments, including lower intrinsic (*r*) and finite rates of increase (*λ*), lower net reproduction (*R*_*0*_) and survival rates, and prolonged generation time. Field studies are required to evaluate and validate the laboratory results of the present study.
